# National Thrift

**Published:** 1889-03-23

**Authors:** 


### National Thrift.

It is to a certain extent taking advantage of your hearers to invite people to a concert, and give them a lecture in the midst of it; it is that powder in the jam which maturity hates as much in the metaphorical, as childhood does in the material sense; and when the said hearers are hospital patients, who are physically unable to get away from advice and information that may be the reverse of pleasing to their ears, it must be admitted that only a good cause will justify such strong measures. But we think that in such a good cause as the inculcation of thrift, Canon Blackley was more than justified in delivering an address to the patients of the Royal Hants County Hospital, when a concert was given for their entertainment in the accident ward of that institution.

We English are not a thrifty nation, which is bad; and we are proud of our extravagance, which is worse. We make a merit of being open-handed, and we are very scornful of certain northern cousins of ours, who are said to keep a sharper look-out on their departing "saxpences" than we. Certainly parsimony is bad, and avarice is detestable, but we do not speak of these, but of modest prudence—the remembrance in the time of youth and strength, when money can be gained and saved, of the night of old age, when no man can work. The fable of the ant and the grasshopper is not a new one, but the moral of it seems hard to grasp; and when it appears from statistics that nearly one half of our people who reach the age of sixty are paupers, it really looks as if we were a nation of grasshoppers.

In no other country is there such a demand for State support in old age as in Britain, poor laws such as ours are unknown elsewhere. Abroad, in France and Germany, where wages are lower, the people recognize the duty of themselves providing for their old age, and display a thoughtfulness and economy almost unknown among our working classes. There are of course many cases in which saving has been impossible, or circumstances have made one call after another on a little hoard; but these do not affect the general fact that our working classes as a whole do not regard it as a duty to make some provision for old age. This bears hardly, not merely on the rich, but on the more thrifty section of the poor. "I do not mind a pin about the rich having to pay," says Canon Blackley, "but where the hardship of the poor law comes in, is that if one of the thrifty of the labouring class is industrious and lays by for his own wants, then from his thrift, his self-denial, and his savings, he has to put his hand in his pocket to pay for the wasteful, the dissolute, and the drunken."

This is bad enough, but perhaps the worst part of the evil is that those who are inclined to idleness feel sure that however lazy and self-indulgent they may be, they will be provided for by other people. "If a man will not work, neither shall he eat," is a severe doctrine, but not an unjust one, and the corollary to it comes in Ruskin's doctrine, that if a man does not earn his dinner he practically steals his dinner—that is, somebody else has to earn it for him. Worst of all is the fact that this improvidence becomes hereditary. Pauper children are likely to be paupers; and now that industrial schools have been long enough established for their workings to be perceived in more than one generation, it is a matter of statistical knowledge that a large proportion of those who were brought up at the public expense—and fitted to earn a decent living, mark that!—leave their children to be educated as they themselves were, at other people's expense. In admitting the necessity, in existing circumstances, for workhouses, industrial schools, and the like, it must not be forgotten that in some cases they have a vicious influence in lessening the feeling of personal and parental responsibility.

Canon Blackley's idea is to establish a system of national insurance, by which all, rich and poor alike, would contribute a small weekly sum, which would give them a right to eight shillings a-week if they were ill, and a pension of four shillings a-week in old age. Some would not claim these pensions—so much the better for those who did; but everyone who contributed would have the satisfactory feeling that he was safe from absolute want, or its alternative of charity. And, we venture to think—in fact, one instance familiar to readers of The Hospital abundantly proves—that the rich are always generously ready to supplement the savings of those who show a desire to help themselves. Gifts, beyond the weekly contributions, would be added to this national insurance fund. But more important even than this provision itself would be the development in our improvident poor of the idea of national thrift.

